# Media Reporting of the 2024 US Preventive Services Task Force Mammography Guideline Update

**DOI:** 10.1001/jamanetworkopen.2026.0040

**Published:** 2026-03-02

**Authors:** Tamar Parmet, Yue Li, Joseph Cappella, Laura D. Scherer, Erika A. Waters, Ashley J. Housten

**Affiliations:** 1 School of Medicine, University of Colorado Anschutz Medical Campus, Aurora; 2 Department of Psychology, University of Colorado Denver; 3 Department of Communication Studies, University of Kansas, Lawrence; 4 Annenberg School of Communication, University of Pennsylvania, Philadelphia; 5 Philadelphia VA Medical Center, Philadelphia, Pennsylvania; 6 Division of Cardiology, University of Colorado Anschutz Medical Campus, Aurora; 7 Division of Public Health Sciences, Department of Surgery, Washington University School of Medicine, St Louis, Missouri

## Abstract

This qualitative study examines whether media coverage of the US Preventive Services Task Force (USPSTF) updated guideline for breast cancer screening also covers the USPSTF’s stance on informed decision-making.

## Introduction

In 2024, the US Preventive Services Task Force (USPSTF) updated its breast cancer screening (BCS) recommendation to biennial screening for women starting at age 40 years,^1^ a shift from its prior guideline emphasizing individualized decision-making for women aged 40 to 49 years.^2^ The USPSTF cited increasing breast cancer incidence among women aged 40 to 49 years and racial disparities as justifications for the change.^1^ Although the updated recommendation contains no explicit language about informed decision-making, the USPSTF has elsewhere emphasized informed choice across all its recommendations.^3^

The media plays a key role in disseminating health information.^4^ As the 2024 BCS guideline does not reiterate the USPSTF’s broader stance on informed decision-making, a potential communication gap exists between the USPSTF’s commitment to informed decision-making and language appearing in the new guideline. We examined whether media coverage of the guideline change has covered both the new recommendation and the USPSTF’s stance on informed decision-making.

## Methods

This qualitative study was a cross-sectional content analysis of digital news covering the 2024 USPSTF BCS guideline. As the data posed no harm to human participants, local ethics review and informed consent were not required in accordance with the Common Rule. The study followed the SRQR reporting guideline.

The Pew Research Center reports the most trusted news outlets by political orientation, with 13 left-leaning, 9 right-leaning, and 1 center-leaning.^5^ We sampled from these outlets, resulting in a total of 23 news outlets (Table 1). We cross-referenced 2 national polls to confirm that the selected outlets were also among those most frequently read in the US in 2024.^6^

**Table 1.** Sampled News Outlets

News site	Political category	Article
**Before guideline change**		
*The New York Post*	Right-center	Diaz A. Breast cancer screenings should begin at 40—not 50: health alert. *New York Post*. May 9, 2023. Accessed April 7, 2024. https://nypost.com/2023/05/09/breast-cancer-screenings-should-begin-at-40-not-50-health-alert/
*The Wall Street Journal*	Right-center	Abbott B. Women should be screened for breast cancer 10 years earlier, guidelines say. *Wall Street Journal*. May 9, 2023. Accessed February 22, 2024. https://www.wsj.com/articles/breast-cancer-screening-from-age-40-would-save-more-lives-experts-say-cf445874?mod=Searchresults_pos1&page=1
*The Washington Examiner*	Right	Severi M. Task force proposes lowering mammogram starting age to 40. *Washington Examiner*. May 9, 2023. Accessed February 5, 2024. https://www.washingtonexaminer.com/news/1358119/task-force-proposes-lowering-mammogram-starting-age-to-40/
Fox News	Right	Rudy M. New breast cancer screening guidelines call for women to start mammograms at age 40. Fox News. May 9, 2023. Accessed January 26, 2024. https://www.foxnews.com/health/new-breast-cancer-screening-guidelines-call-women-start-mammograms-age-40
*The Epoch Times*	Right	Stieber Z. Breast cancer screenings should start 10 years earlier, new guidelines day. *Epoch Times*. May 9, 2023. Accessed February 5, 2024. https://www.theepochtimes.com/health/breast-cancer-screenings-should-start-10-years-earlier-new-guidelines-say-5252818
*The Epoch Times*	Right	Suttie E. Why the new breast cancer screening guidelines may not save more women. *Epoch Times*. May 22, 2023. Accessed February 8, 2024. https://www.theepochtimes.com/health/why-the-new-breast-cancer-screening-guidelines-wont-save-more-women-5262960
NewsMax	Right	Annual mammograms starting at 40 saves lives. NewsMax. February 20, 2024. Accessed March 22, 2024. https://www.newsmax.com/health/health-news/mammogram-guidelines-screening/2024/02/20/id/1154297/
NewsMax	Right	Panel now recommends mammograms start at age 40. NewsMax. May 9, 2023. Accessed March 1, 2024. https://www.newsmax.com/health/health-news/mammogram-women-age/2023/05/09/id/1119150/
*Forbes*	Right-center	Awan O. New breast cancer screening guidelines are a step in the right direction, but one more step is needed. *Forbes*. May 10, 2023. Accessed March 12, 2024. https://www.forbes.com/sites/omerawan/2023/05/10/new-breast-cancer-screening-guidelines-are-a-step-in-the-right-direction-but-one-more-step-is-needed/?sh=5159c37067f5
BBC News	Left-center	Bailey C. US health panel releases new breast cancer screening guidelines. BBC News. May 9, 2023. Accessed February 2, 2024. https://www.bbc.com/news/world-us-canada-65539932
CNN	Left-center	Howard J. Women should start screening for breast cancer at age 40 instead of 50, health task force says in draft recommendation. CNN. May 9, 2023. Accessed January 26, 2024. https://www.cnn.com/2023/05/09/health/breast-cancer-screening-age-40-uspstf/index.html
NBC News	Left-center	Bendix A. Screen all women for breast cancer at 40, instead of 50, new guidelines say. NBC News. May 9, 2023. Accessed March 1, 2024. https://www.nbcnews.com/health/womens-health/breast-cancer-guidelines-start-screenings-age-40-rcna83355
MSNBC	Left	Boone L. Avoiding a mammogram? high heels and waxing are more painful. MSNBC. May 15, 2023. Accessed March 12, 2024. https://www.msnbc.com/know-your-value/health-mindset/u-s-panel-lowers-recommended-age-women-get-mammograms-n1305096#
*The New York Times*	Left-center	Rabin RC. When should women get regular mammograms? at 40, U.S. panel now says. *New York Times*. May 9, 2023. Accessed March 1, 2024. https://www.nytimes.com/2023/05/09/health/breast-cancer-mammograms.html
*The New York Times*	Left-center	Rabin RC. New mammogram advice: what to know. *New York Times*. May 10, 2023. Accessed April 7, 2024. https://www.nytimes.com/2023/05/09/health/mammograms-frequency-women.html
*The New York Times*	Left-center	Golshan M. I’m a breast cancer survivor. here’s what I think of the new screening guidelines. *New York Times*. May 16, 2023. Accessed April 9, 2024. https://www.nytimes.com/2023/05/16/opinion/breast-cancer-screening.html
*The New York Times*	Left-center	Blum D. What 40-somethings should know about breast cancer. *New York Times*. May 18, 2023. Accessed April 12, 2024. https://www.nytimes.com/2023/05/18/well/live/breast-cancer-risk-prevention.html?searchResultPosition=2
PBS	Left-center	Associated Press. Start mammograms at 40, not 50, U.S. health panel recommends. PBS News. May 9, 2023. Accessed March 7, 2024. https://www.pbs.org/newshour/health/start-mammograms-at-40-not-50-u-s-health-panel-recommends
Reuters	Center	Lapid N. US panel drops mammography screening age back to 40. Reuters. May 9, 2023. Accessed February 9, 2024. https://www.reuters.com/world/us/us-panel-calls-breast-cancer-screening-start-age-40-2023-05-09/
*USA Today*	Left-center	Hassanein N. Breast cancer screening should start at age 40—10 years earlier than previous advice, group says. *USA Today*. May 9, 2023. Accessed March 12, 2024. https://www.usatoday.com/story/news/health/2023/05/09/breast-cancer-mammograms-should-begin-at-age-40-new-guidelines-say/70185090007/
*The Washington Post*	Left-center	Wen LS. Mammograms at age 40? yes, please. *Washington Post*. May 18, 2023. Accessed February 5, 2024. https://www.washingtonpost.com/opinions/2023/05/18/breast-cancer-screening-mammogram-age/
*The Washington Post*	Left-center	Bever L. Health panel recommends women get screening mammograms at age 40. *Washington Post*. May 9, 2023. Accessed February 2, 2024. https://www.washingtonpost.com/wellness/2023/05/09/mammogram-age-40-breast-cancer-screening/
**After guideline change **		
*Forbes*	Right-center	Gleason C. Breast cancer screenings should start at age 40, U.S. task force says. *Forbes*. April 30, 2024. Accessed June 13, 2024. https://www.forbes.com/sites/caileygleeson/2024/04/30/breast-cancer-screenings-should-start-at-age-40-a-decade-earlier-than-before-task-force-says/?sh=1896f19b5fb9
Fox News	Right	Rudy M. Breast cancer mammogram screenings should start at age 40 instead of 50, says health task force. Fox News. April 30, 2024. Accessed June 26, 2024. https://www.foxnews.com/health/breast-cancer-mammogram-screenings-should-start-40-instead-50-says-health-task-force.print
*The Epoch Times*	Right	Dahnke AC. National task force lowers mammogram screening age to 40. *Epoch Times*. April 30, 2024. Accessed June 26, 2024. https://www.theepochtimes.com/health/national-task-force-lowers-mammogram-screening-age-to-40-post-5640609?welcomeuser=1
*The Washington Post*	Right	Bever L. Confused by new mammogram guidelines? here’s what to know. *Washington Post*. May 8, 2024. Accessed June 17, 2024. https://www.washingtonpost.com/wellness/2024/05/08/screening-mammograms-insurance/
NewsMax	Right	Panel: mammograms should start at age 40. NewsMax. April 30, 2024. Accessed June 12, 2024. https://www.newsmax.com/health/health-news/mammogram-breast-cancer-screening-guidelines/2024/04/30/id/1162938/
Reuters	Center	Niasse A. Breast cancer screening should begin at age 40, US panel says. Reuters. April 30, 2024. Accessed June 26, 2024. https://www.reuters.com/business/healthcare-pharmaceuticals/breast-cancer-screening-should-begin-age-40-us-panel-says-2024-04-30/
PBS	Left-center	Johnson CK. Mammograms should start at 40 to address rising breast cancer rates, U.S. panel says. PBS News. April 30, 2024. Accessed June 26, 2024. https://www.pbs.org/newshour/health/mammograms-should-start-at-40-to-address-rising-breast-cancer-rates-u-s-panel-says
*Today*	Left-center	Jacoby S. Breast cancer screening should start at 40, new expert recommendations say. *Today*. May 1, 2024. Accessed June 14, 2024. https://www.today.com/health/breast-cancer/new-breast-cancer-screening-guidelines-2024-rcna149381
*USA Today*	Left-center	Alltucker K. Task force says this is when women should begin breast cancer screening, get mammograms. *USA Today*. April 30, 2024. Accessed June 26, 2024. https://www.usatoday.com/story/news/health/2024/04/30/women-breast-cancer-screening-40s-guidelines/73500806007/#
*The New York Times*	Left-center	Wong J, Nicholson W. In reversal, expert panel recommends breast cancer screening at 40. *New York Times*. April 30, 2024. Accessed June 1, 2024. https://www.nytimes.com/2024/04/30/health/breast-cancer- mammography-ultrasound.html
CNN	Left-center	Howard J. Task force updates guidance for breast cancer screenings for women 40 and older. CNN. May 1, 2024. Accessed June 5, 2024. https://www.cnn.com/2024/04/30/health/breast-cancer-mammograms-earlier-recommendations/index.html
NBC News	Left	Bendix A. Get screened for breast cancer starting at age 40, new recommendations say. NBC News. April 30, 2024. Accessed June 7, 2024. https://www.nbcnews.com/health/womens-health/breast-cancer-screening-start-40-new-recommendation-rcna149777/

Four search terms were used to identify articles: *breast cancer screening*, *mammography(ies)*, *USPSTF, guideline(s)*, and *breast cancer screening guideline(s)*. Searches were limited to 2 months after the draft and final guideline were released (May to July 2023 and 2024).

A codebook was developed and revised iteratively to categorize data collected. Consensus coding categories included discussion of basic information about the guideline change, the rationale for the change, BCS benefits (lives saved) or harms (eg, overdiagnosis, false-positive results), and informed decision-making. Descriptive statistics were summarized using SPSS, version 30 (IBM Corp).

## Results

Thirty-five articles were included. No discernable differences by news outlet political leaning were observed. All 35 articles discussed the recommended screening age and frequency (Table 2). Most articles discussed USPSTF’s rationale for guideline change, with 26 (74.3%) highlighting the increased incidence of breast cancer and 28 (80.0%) discussing racial disparities. Thirty-three articles (94.3%) described BCS benefits, and 27 (77.1%) explicitly highlighted breast cancer deaths prevented. False-positive results (25 articles [71.4%]) and follow-up biopsies (19 articles [54.3%]) were the harms most frequently cited. Only 7 articles (20.0%) mentioned overdiagnosis.

**Table 2.** Exemplar Quotes

Category	Articles, No. (%)	Exemplar quotes
Articles that mention the frequency of screening	35 (100)	“Women are now advised to get a mammogram every other year starting at age 40 and until age 74, according to new recommendations from the US Preventive Services Task Force.” (Howard 2024)
Articles that discussed the rationale for the guideline change		
Increased incidence of breast cancer	26 (74.3)	“We make this new updated recommendation because the latest science clearly shows that starting at age 40 and obtaining a mammogram every other year until age 74 can further reduce deaths in breast cancer.” (Howard 2024)
Lives saved	27 (77.1)	
Racial disparities	28 (80.0)	
Articles that discussed informed decision-making		
All	26 (74.3)	“A 2016 update from the USPSTF had said women could start screening at age 40 if they and their doctors so desired, but that update led “to widespread confusion for both physicians and patients.” (Niasse 2024) “Leading breast cancer organization Susan G. Komen said in a statement it ‘generally supports’ the updated recommendations but urged patients to discuss personalized approaches with their health care providers.” (Gleeson 2024)
Related to old guideline	23 (65.7)	
Related to new guideline	5 (14.3)	
Articles that discussed the benefits of screening	33 (94.3)	“This new recommendation will help save lives and prevent more women from dying due to breast cancer.” (Severi 2024)
Articles that discussed the harms of screening		
Any	30 (85.7)	“Getting screened every year also significantly increases the likelihood for potential harms, Nicholson says. Those harms include false-positive results, which can result in unnecessary biopsies, among other treatments and tests.” (Jacoby 2024) “Frequent screening can itself cause harm, leading to unnecessary biopsies that cause anxiety and treatment for slow-growing cancers that would never have been life-threatening, researchers have found.” (Rabin 2023)
False-positive results	25 (71.4)	
Overdiagnosis	7 (20.0)	
Financial harms	2 (5.7)	
Psychological harms (eg, anxiety)	15 (42.9)	
Physical harms (eg, follow-up biopsy)	19 (54.3)	
Article makes a statement that the benefits of screening outweigh the harms	12 (34.3)	“‘The benefits of screening, such as halting the natural progression of cancer, increasing treatment options that are well-tolerated and saving lives, far outweigh the risks of false positives, which can be resolved with additional imaging and biopsies,’ Barofsky added.” (Rudy 2023)
Article questioned USPSTF decision to recommend biennial screening over annual screening	12 (34.3)	“Experts say the guidelines are a leap in the right direction but should go further to advise women to be screened annually.” (Hassanein 2023)

Abbreviation: USPSTF, US Preventive Services Task Force.

Twenty-six articles (74.3%) mentioned informed decision-making, with 23 (65.7%) mentioning informed decision-making in reference to the previous USPSTF guideline. Only 5 (14.2%) discussed informed decision-making in reference to the new guideline, encouraging patients to engage in conversations with their clinicians to create a personalized screening plan. No article mentioned the USPSTF’s broader stance on informed decision-making.^3^ Exemplar quotes are shown in Table 2.

## Discussion

The USPSTF presented a strong case for informed decision-making across all its recommendation levels, and since consensus is lacking on BCS initiation age and frequency across guidelines (eg, USPSTF, American Cancer Society), it remains critical to promote informed decisions for screening. The media plays a pivotal role in this process by creating context for patients to apply their preference and values to their health care decisions. This qualitative study found that news outlets consistently conveyed the basic details of the 2024 USPSTF BCS guideline, but coverage rarely addressed overdiagnosis and seldom discussed informed decision-making in referenced to the new guideline. A limitation of this study was that only web-based, written materials were included. Therefore, some information accessed by the public may have been missed.

## Notes

Supplement.
Data Sharing Statement
