# Supplementary material for: Media Reporting of the 2024 US Preventive Services Task Force Mammography Guideline Update
Source: JAMA Netw Open. 2026 Mar 2;9(3):e260040. doi: 10.1001/jamanetworkopen.2026.0040 (PMC12954536; doi:10.1001/jamanetworkopen.2026.0040)
Supplement: Supplement. — Data Sharing Statement [file jamanetwopen-e260040-s001.pdf]

Data Sharing Statement

Parmet. Reporting the 2024 US Preventive Services Task Force Mammography Guideline Update. *JAMA Netw Open*. Published March 02, 2026. doi:10.1001/jamanetworkopen.2026.0040

Data
Data available: No

Additional Information
Explanation for why data not available: If readers would like a copy of the qualitative coded database we are happy to share it.
